# Temporal Response Function-Driven Representational Similarity Analysis for Speech Perception Decoding with MEG and EEG

**DOI:** 10.3390/biology15131028

**Published:** 2026-06-28

**Authors:** Changzeng Liu, Yu Guo, Jin Ding, Ling Li, Yuyu Ma, Xiaolin Ning

**Affiliations:** 1 Key Laboratory of Ultra-Weak Magnetic Field Measurement Technology, Ministry of Education, School of Instrumentation and Optoelectronic Engineering, Beihang University, Beijing 100191, China; by2143115@buaa.edu.cn (C.L.); by2354105@buaa.edu.cn (J.D.); amber02@buaa.edu.cn (L.L.); 2 Hefei National Laboratory, Hefei 230088, China; 3 College of Engineering and Computer Science, Syracuse University, Syracuse, NY 13244, USA; yguo1211@gmail.com; 4 State Key Laboratory of Smart Power Distribution Equipment and System, School of Electrical Engineering, Hebei University of Technology, Tianjin 300401, China; mayuyu@hebut.edu.cn; 5 Shandong Key Laboratory for Magnetic Field-Free Medicine and Functional Imaging, Shandong University, Jinan 250014, China; 6 State Key Laboratory of Traditional Chinese Medicine Syndrome, Guangzhou University of Chinese Medicine, Guangzhou 510006, China

**Keywords:** brain, OPM-MEG, EEG, decoding, speech perception, temporal response function, representational similarity analysis

## Abstract

Speech perception relies on distributed neuronal populations, yet traditional decoding often utilizes static strategies that overlook inherent temporal dependencies and dynamic regulation. Therefore, we introduce the concept of system identification into multivariate decoding. By modeling brain response characteristics through time-lagged regression between speech stimuli and neural responses, we propose a temporal response function-based representational similarity analysis method (TRF-RSA). This method models the dynamic time-lag mapping from continuous stimulus features to neural responses, effectively separating stimulus-driven coherent activity from high-dimensional noise. More importantly, it elevates the analytical perspective from static comparisons of raw signals to dynamic trajectories in weight space. We conducted an auditory experiment and incorporated high spatiotemporal resolution optically pumped magnetometer magnetoencephalography magnetoencephalography (OPM-MEG) with electroencephalography (EEG). The results showed that TRF-RSA significantly enhanced the pattern similarity between speech sounds and the ability to discriminate between pattern differences. Furthermore, it revealed stronger similarities elicited by biological vocalizations, indicating a preference in the brain for these species-specific sounds. Source localization results not only confirmed the classical speech perception network but also revealed activation in limbic and deep brain regions. By modeling the relationship between stimulus features and neural responses, TRF-RSA dynamically quantified the spatiotemporal patterns of stimulus-driven neural activity, improving the sensitivity of representational pattern decoding during the encoding process. These findings suggest that this method is a sensitive neuroimaging tool that not only advances our understanding of the spatiotemporal dynamics of speech processing but also provides a new reference for population dynamics research.

## Simple Summary

Understanding how the human brain processes and comprehends various sounds remains a major challenge in modern science. Traditional multivariate analysis methods often employ static strategies and overlook the temporal dependencies of neural responses. Therefore, our primary objective was to integrate system identification principles into multivariate pattern analysis. By modeling the relationship between stimulus features and neural responses, we developed a dynamic representation analysis method that successfully separated meaningful sound-driven brain activity from background neural noise. The results demonstrated that this approach offered much higher sensitivity in decoding different categories of sounds, revealing that the human brain showed a distinct preference for biological sounds. Furthermore, it uncovered a much wider auditory network than previously thought, involving not just traditional auditory areas but also limbic and deep brain structures. This work deepens our understanding of the spatiotemporal dynamics of sound processing and highlights the potential of this method for objective clinical assessment and diagnosis.

## 1. Introduction

Fundamentally, human cognitive functions and behaviors are realized through complex spatiotemporal activity patterns in the brain [1]. Contemporary neuroscience emphasizes that cognitive function relies on distributed networks of cortical sites which interact through complex spatiotemporal patterns [2]. External stimuli evoke complex neural signals that manifest as specific spatiotemporal patterns of coordinated neuronal population activity, interacting dynamically across distributed cortical networks. During this process, the human brain rapidly receives and integrates the spatial and temporal characteristics of sensory input across multiple levels and different time scales. Specific activation patterns correspond to specific representational content, collectively constituting a high-dimensional coding space [3]. These spatiotemporal patterns characterize not only the instantaneous activation states of specific brain regions but also their dynamic evolution over time. Consequently, they reflect the intrinsic communication mechanisms and information representation underlying brain function [4,5]. Among various forms of cognition, speech perception serves as a representative process that embodies this capacity for multi-scale, high-dimensional spatiotemporal integration.

Speech conveys both social and biological information, serving not merely as a linguistic medium but also transmitting intentions, emotions, gender, and identity [6]. Efficient speech perception requires the brain to rapidly and accurately extract fine-grained acoustic features and encode them into abstract representational patterns. During continuous speech perception, the auditory cortex executes a fine-grained feature decomposition of the acoustic signals. Ultimately, external physical acoustic stimuli are successfully transformed into perceptual experiences, semantic comprehension, and high-level cognitive representations [7,8]. Failures in this rapid processing are often linked to clinical deficits. While the significance of speech perception is well established, the precise spatiotemporal dynamics and high-dimensional nature of its underlying neural representational processes remain relatively limited. Inspired by this idea, recent analyses of neural recordings and functional imaging data have increasingly focused on the activity patterns of multiple neuronal populations, aiming to better understand how the brain encodes information through complex activation patterns.

In recent years, a variety of brain activity recording modalities have been developed. Recent advances in quantum precision measurement techniques have promoted the development of magnetic field detectors. Optically Pumped Magnetometer Magnetoencephalography (OPM-MEG) is an emerging magnetic field detection technology that allows sufficient sampling of rapidly changing neuronal populations. This technique provides higher quality data, more uniform coverage, motion robustness, and lower system complexity [9], serving as an important tool for neuroscience and clinical disease research.

Meanwhile, an increasing number of studies are shifting focus towards decoding neural representations by analyzing patterns of activation across multiple voxels rather than modeling individual voxels [3]. Representational similarity analysis (RSA), as an emerging multivariate analysis technique, has been widely applied across various domains in neuroscience [10]. This approach enables the simultaneous consideration of multiple features, effectively measuring the similarity of activity patterns across different conditions. It offers advantages in cross-task, cross-modal, and cross-population analyses [11], and is increasingly favored by researchers.

While RSA can characterize the geometric features of activation patterns induced by different stimuli in a common space through a representational similarity matrix (RSM), it still faces several limitations. First, this method often constructs a similarity metric based solely on neural signals [12]. As raw signals contain task-irrelevant components and noise, this approach suffers from insufficient sensitivity in neural pattern decoding. More importantly, neurons and synapses exhibit rich temporal dependencies: the relationship between stimuli and neural responses is neither instantaneous nor independent, with both exhibiting temporal autocorrelations [13]. This intrinsic property implies that neural encoding is modulated by both incoming stimuli and the internal state of neural networks, which may be driven either by external stimulus structures or internal dynamics [14]. Therefore, traditional RSA, as a static analysis strategy, inevitably produces blurred results.

To capture the dynamic evolution of neural representations, recent studies have attempted to employ methods such as sliding time windows [15] or dynamic time warping [16] to construct time-varying representational similarity matrices. However, these strategies remain susceptible to contamination from environmental noise and endogenous physiological artifacts. Crucially, such methods fail to effectively capture the time-lagged dependencies between external stimuli and brain activity, thereby limiting temporal resolution and representational sensitivity. To overcome these limitations, the primary objective of this study is to integrate system identification principles into multivariate pattern analysis. By constructing a novel dynamic representational similarity analysis framework based on temporal response functions (TRF-RSA), this approach aims to enhance the sensitivity of existing methods in extracting high-dimensional, dynamic neural representations.

Therefore, this study modeled brain response characteristics using speech stimulation and neural signals, proposing a temporal response function-based representational similarity analysis framework (TRF-RSA). The TRF, derived from system identification principles, offers a solution by explicitly modeling the dynamic, time-lagged mapping from a continuous stimulus feature to the continuous response. By quantifying the weighted contribution of stimulus features to the neural response across a series of time lags, the TRF captures the temporal dynamics and integration processes required for transforming stimulus input into observed neural activity [17]. This dynamic modeling addresses the static limitation, as it precisely captures the crucial temporal dependencies between the evolving speech stimuli and the subsequent neural activity. Moreover, it effectively extracts stimulus-specific representations from background noise, focusing on the neural activity coherently driven by the input stimulus [18,19]. We hypothesize that combining this dynamic model with geometric analysis techniques may provide a more physiologically relevant and stable feature space, thus addressing the limitations mentioned above.

## 2. Method

This study is structured as follows. First, we conducted a speech discrimination experiment, stimulating the brain’s speech processing network by presenting a large variety of different sound stimuli (6 categories, a total of 90 stimuli). We presented speech sounds to 16 participants and recorded OPM-MEG and EEG data during the experiment. Neural signals robustly track the amplitude envelope of input stimuli, which is a key cue for speech comprehension [20]. Therefore, we extracted the envelopes across all speech stimuli and subsequently constructed TRFs for all channels by modeling the relationship between these envelope features and the recorded neural signals. Furthermore, we constructed RSMs to reveal the dynamically evolving neural response pattern similarity (NRPS) during speech perception. We subsequently computed NRPS within specific stimulus categories, enabling systematic comparisons between TRF-RSA and traditional RSA (denoted Tra-RSA) in their sensitivity for pattern similarity. Finally, we showed the source localization results to investigate the anatomical origins of NRPS dynamics.

### 2.1. Time-Lagged Regression

**Figure 1:**
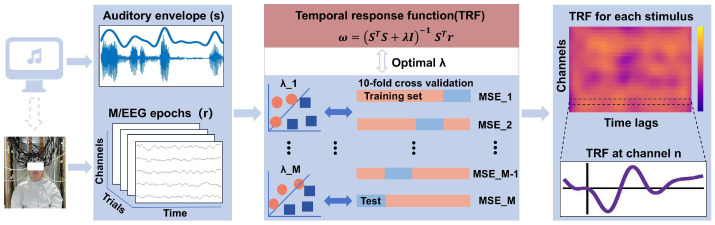
Estimation of the TRF. Combining the speech envelope and M/EEG signals, the TRF model was constructed through time lag regression.

This study employed a time-lagged regression mode. Based on the trial-averaged M/EEG responses and the speech envelope, this model was utilized to establish a mapping relationship between the acoustic stimulus features and the neural response. Specifically, we hypothesized that the observed neural response was the result of the convolution between the speech envelope and a set of time-lagged coefficients. In this process, the root mean square error between the actual M/EEG signals and their estimated values was minimized (Figure 1).

We first calculated the Hilbert transform of the original speech waveform x(t). The speech envelope refers to the slowly varying contour of the amplitude of a speech signal over time. It carries critical syllabic and rhythmic structures and serves as a crucial cue for speech comprehension [21,22]. With the speech envelope used as the input, the time-domain response function derived via linear time-lagged regression modeling is the envelope-based TRF. We then extracted its absolute value and applied a 0.6 power-law compression [23]. Finally, the signal was downsampled to 128 Hz to obtain the broadband amplitude envelope s(t):

(1) $$\begin{array}{c} s\left(t\right)\;=\;{\left|H\left[x(t)\right]\right|}^{0.6} \end{array}$$

Assuming the M/EEG system consists of *N* recording channels, r(t,n) represents the trial-averaged neural response at time point *t* on channel *n*. s(t) is the speech envelope at time *t*, which has an unknown specific TRF in this channel, namely ω(τ,n). It is the transformation (TRF) of the stimulus at time lag τ on channel *n*, then the response model can be expressed as:

(2) $$\begin{array}{c} r\left(t,\;n\right)\;=\;\sum_{\tau }\omega \left(\tau ,\;n\right)s\left(t\;-\;\tau \right)\;+\;\varepsilon \left(t,\;n\right) \end{array}$$

where ε(t,n) is the residual response of the channel and s(t−τ) is the speech envelope at a lag τ, spanning from −100 ms pre-stimulus to 1000 ms post-stimulus. This model was estimated by considering multiple stimulus-response time lags τ. The TRF was obtained by minimizing the root mean square error between the actual neural response and the predicted response from convolution. During model estimation, the temporal window was [−100 − 10, 1000 + 10] ms to avoid edge artifacts, resulting in a full set of cross-validated TRF estimates ω(τ,n).

(3) $$\begin{array}{c} \min\varepsilon \left(t,\;n\right)\;=\;\sum_{t}{\left[r\left(t,\;n\right)\;-\;\hat{r}\left(t,\;n\right)\right]}^{2} \end{array}$$

where $\hat{r}\left(t,n\right)$ is the neural response estimated by the TRF model:

(4) $$\begin{array}{c} \hat{r}\left(t,\;n\right)\;=\;\sum_{\tau }\omega \left(\tau ,\;n\right)s\left(t\;-\;\tau \right) \end{array}$$

Given the speech features of multiple time lags had autocorrelations and the regressor dimension was large, we used Tikhonov-regularized regression to estimate the response coefficient and suppress overfitting [24]. In practice, the system weight vector ω was solved by reverse correlation and least squares with regularization [25].

(5) $$\begin{array}{c} \omega \;=\;{\left(S^{T}S\;+\;\lambda I\right)}^{-1}S^{T}r \end{array}$$

where the matrix *S* is the lagged time series of the stimulus matrix *s*. *r* is the neural signals, and *I* is the identity matrix. λ is the regularization parameter used to prevent overfitting. For each participant and each electrode separately, TRF was fitted using regularized linear regression to establish a mapping relationship between neural signal amplitude and time-lagged acoustic envelope representations. Concretely, candidate values of the regularization parameter λ were set in the logarithmic space within the range $10^{-12}$ to $10^{12}$, with a step size of power of 10. Through 10-fold cross-validation with repeated training and validation procedures, the λ that minimized the mean normalized root mean squared error was automatically selected. The trained TRF weight ω represent the response amplitude at different time delays in response to a unit change in the given stimulus feature. Continuous changes in neighboring time-lag points indicate the dynamic processing of the auditory perception system. Before formal TRF analysis, both the raw neural signals and acoustic envelopes were normalized by their respective standard deviations.

### 2.2. Representational Similarity Analysis

In this study, RSA was employed to project neural responses onto a common representational space. Within this space, brain activity evoked by a specific speech stimulus is represented as a corresponding neural response pattern vector, with different speech stimuli forming distinct distributions in the high-dimensional space. RSMs systematically quantify the pairwise similarity between these pattern vectors, where the resultant geometric structure indirectly reflects the organizational properties of the neural representation.

**Figure 2:**
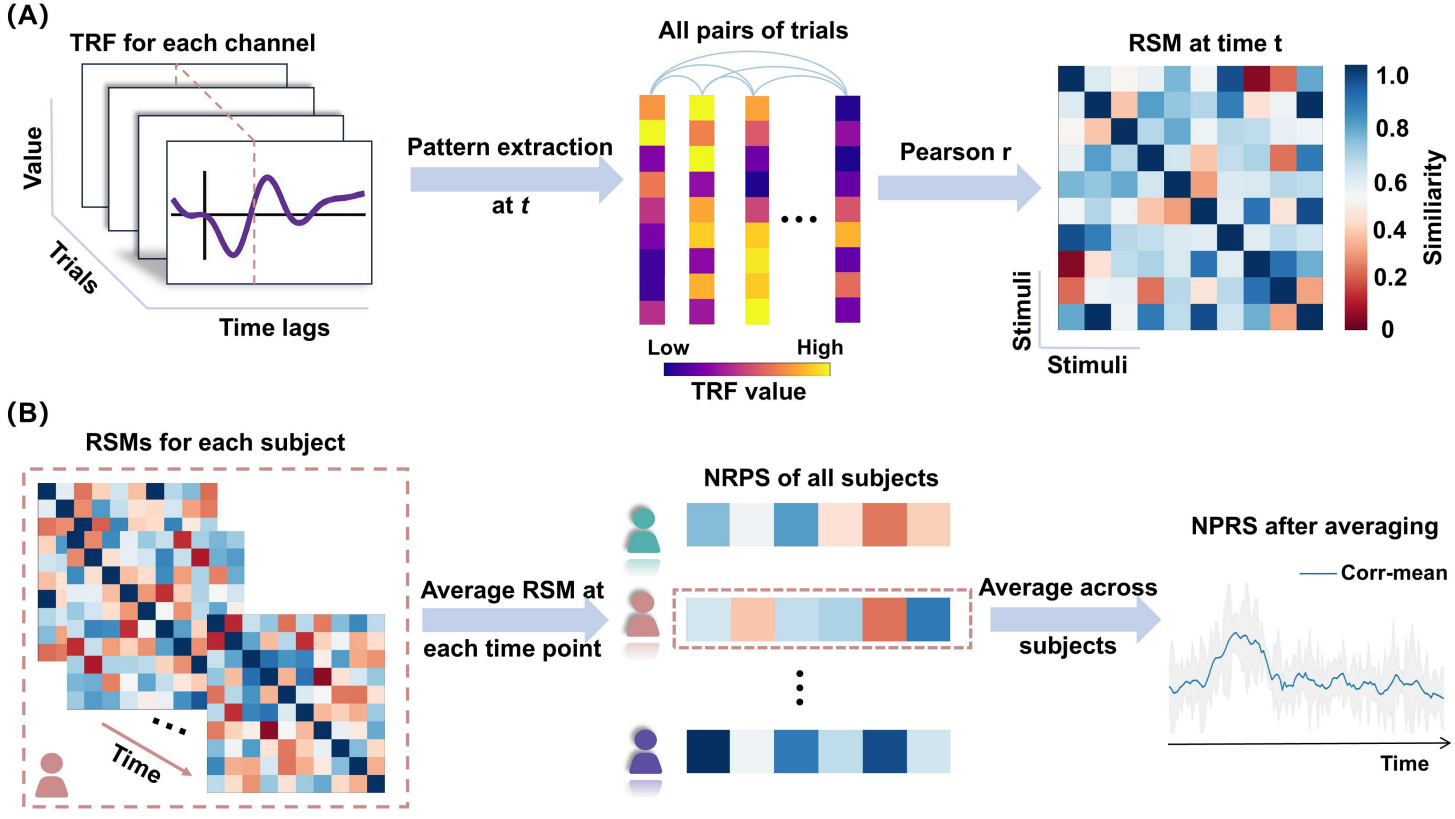
Schematic representation of the similarity analysis. (**A**) Calculation of the RSM at a specific moment. The TRF amplitudes of all channels at the same moment represented the neural response pattern vectors of the brain to the corresponding stimulus. The Pearson correlation coefficient was used to calculate the response pattern similarity between stimulus pairs. This value was the value in the RSM matrix. (**B**) Neural response pattern similarity. The pattern similarity of all possible pairwise stimulus pairs was calculated at each time point, resulting in a series of RSMs over time. The mean of the off-diagonal elements in the lower left corner of each matrix was calculated and further averaged across participants.

RSM is a diagonally symmetric square matrix (Figure 2A). Its dimension is the number of experimental stimuli, and its row/column order corresponds to the stimulus index. The off-diagonal elements reflect the pattern similarity between different stimulus pairs. This geometric structure reveals the interrelationships between neural activity evoked by different stimuli. We averaged the off-diagonal elements in the lower left corner of the matrix. RSMs across all experimental blocks and participants were averaged to obtain a population-level representation of the dynamics of pattern similarity (Figure 2B). Specifically, given the sensitivity of the RSM and subsequent NRPS to outliers and noise [12], the computational basis and input data for Tra-RSA and TRF-RSA were identical, which were both derived from the neural signals of each specific auditory stimulus averaged over 9 repeated trials. For each time point, spatial activation patterns were obtained by vectorizing multi-channel TRFs into neural pattern vectors. Subsequently, RSM was constructed by computing Pearson correlation coefficients (Corr) between all stimulus pairs (where Corr = 1 indicates perfectly correlated neural response patterns, and Corr = 0 indicates no correlation). In Tra-RSA, the NRPS is computed directly within the preprocessed signal space. Specifically, at each time point, spatial activation pattern vectors are obtained by vectorizing the multi-channel, trial-averaged neural signals. Subsequently, correlation coefficients between all pairs of stimuli are calculated for each time point to construct the RSM. This contamination affects the true representational similarity between different stimulus categories, leading to a distortion of the representational space’s geometric structure. Furthermore, as speech is a continuously evolving dynamic stimulus, conventional approaches typically average activity over a broad time window. This static analysis strategy overlooks the fine temporal evolution of speech features and the time-dependent nature of the neural system. Consequently, this blurs the true geometric structure of the neural representations, thus affecting pattern similarity. In contrast, since TRF-RSA transforms *r* through ω, when calculating TRF-RSA:

(6) $$\begin{array}{c} {\mathrm{Corr}}_{\mathrm{TRF}-\mathrm{RSA}}\;=\;\frac{\mathrm{cov}\left(\omega_{1},\;\omega_{2}\right)}{\sigma_{\omega_{1}}\sigma_{\omega_{2}}} \end{array}$$

$\omega_{1}$ and $\omega_{2}$ are the TRF weights under two stimuli. $\mathrm{cov}\left(\omega_{1},\omega_{2}\right)$ represents the covariance of $\omega_{1}$ and $\omega_{2}$. $\sigma_{\omega }$ represents the standard deviation of ω. TRF-RSA offers significant advantages by transforming the input into ω. First, these weights specifically extract neural activity related to the stimulus envelope, effectively separating neural activity unrelated to speech. This improves the signal-to-noise ratio of the representation space, enhancing the accuracy of similarity calculations. Second, by precisely matching and aligning continuous envelope features with neural responses over time, TRF captures the temporal dynamics and integration processes required from stimulus input to cortical response. Therefore, TRF-RSA can more fundamentally and accurately reflect the encoding characteristics of the brain for specific speech, elevating the analysis from a static comparison of signal intensity to a comparison of dynamic encoding features.

## 3. Experiment

### 3.1. Participants

This study recruited 16 healthy, right-handed, native Chinese-speaking adults, including 10 males (27.85±1.73 years) and 6 females (27.43±1.35 years). None of the participants had a history of congenital developmental disorders, hearing impairments, neurological or psychiatric disorders. Informed consent was obtained from all participants before the experiment, and the study was formally approved by the Ethics Committee of Beihang University (No. BM20240174).

### 3.2. Stimuli and Tasks

**Figure 3:**
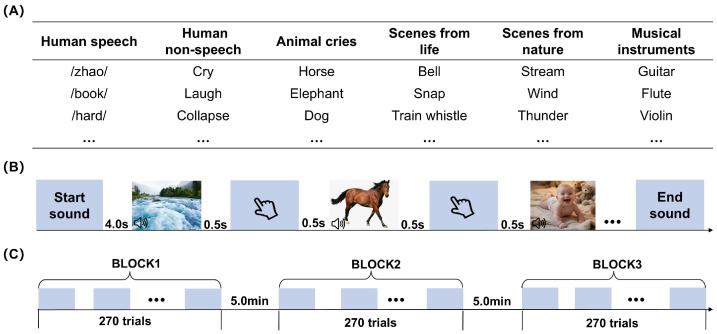
Experimental stimuli and paradigm. (**A**) Example sound stimuli. There were 6 sound categories, each with 15 stimulus samples. (**B**) Experimental paradigm. After each stimulus presentation, participants performed the sound discrimination task by pressing key. (**C**) Block design. Each participant performed 3 blocks, with each sound randomly repeated 3 times, for a total of 810 trials.

Building upon prior research, we established 6 categories of sound materials: Human speeches (HS), Human non-speeches (HNS), Animal cries (AC), Scenes from life (SL), Scenes from nature (SN), Musical instruments (MI), totaling 90 sound stimuli. Figure 3A listed some examples of each type of sound. Stimuli were sampled at 44,100 Hz and lasted approximately 0.8 s. They were normalized to the same amplitude using the sound processing software Praat (version 6.4.07, University of Amsterdam, Amsterdam, The Netherlands; https://www.fon.hum.uva.nl/praat/ (accessed on 23 June 2026)). Normalized stimuli were controlled by MATLAB (R2024a, MathWorks, Natick, MA, USA) and played using Psychophysics Toolbox 3.0. Each sound was randomly repeated 9 times, for a total of 810 trials. However, it is worth noting that although the acoustic stimuli were strictly normalized for basic features (such as amplitude, frequency contours, and duration) using Praat, other or higher-order acoustic characteristics were not normalized.

Stimuli were presented to the participants via insertable silicone air tubes. Before the experiment, the sound intensity was adjusted to a comfortable listening level (65 dB SPL). As shown in Figure 3B, participants were instructed to remain relaxed while performing a forced-choice task to categorize sounds as either animate or inanimate. The experiment employed a block design (Figure 3C). At the beginning of the experiment, a “start cue tone” played, instructing participants on precautions during the experiment. After a 4.0 s interval, the stimuli were presented. After each stimulus, there was a 0.5 s response interval, followed by a “key-press tone”. Upon hearing the tone, participants were instructed to press a button on the feedback box to indicate whether they heard an animate sound (HS, HNS, AC; left button) or an inanimate sound (SL, SN, MI; right button), ensuring their attention throughout the experiment. Subsequently, the participant pressed the key for 0.5 s before the program played the next stimulus. Each block lasted approximately 9 min, and each participant performed 3 blocks. Within each block, all stimuli were presented 3 times in a randomized order. There was a 5 min break between blocks, after which the participant was verbally confirmed of their status and the start of the next block.

### 3.3. Experimental System

**Figure 4:**
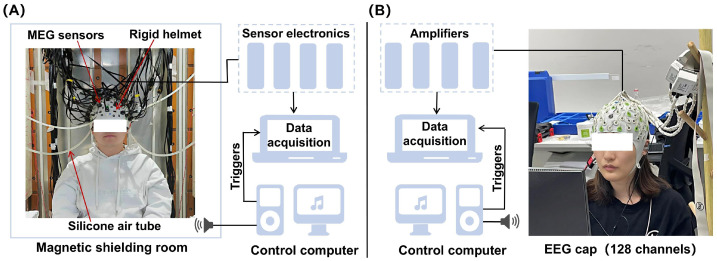
Experimental system. (**A**) The participant wore a rigid helmet equipped with sensors for OPM-MEG data acquisition. (**B**) The participant wore an EEG cap for EEG data acquisition.

We constructed an OPM-MEG system using 36 QuSpin (Quspin Inc., Louisville, CO, USA) second-generation zero-field magnetometers (QZFM Gen2). Each sensor has a sensitivity better than $15\;\mathrm{fT}/\sqrt{\mathrm{Hz}}$ and measures 12.4 mm × 16.6 mm × 24.4 mm. The magnetometer array was stably positioned within a 4 m × 3.5 m × 3 m magnetically shielded room using a 3D-printed rigid helmet (Figure 4A). The central residual magnetic field in this shielded environment was less than 5 nT, effectively isolating the system from ambient magnetic interference.

EEG signals were recorded using a BrainCap electrode array (128-channel configuration, Brain Products GmbH, Gilching, Germany). Electrodes were positioned according to the standardized 10–20 international placement system (Figure 4B). During the experiment, all channel impedances were maintained below 30 kΩ, and “FCz” was set as the online reference channel. Real-time data were filtered online within the 0.01–250 Hz range.

### 3.4. Preprocessing

**Figure 5:**
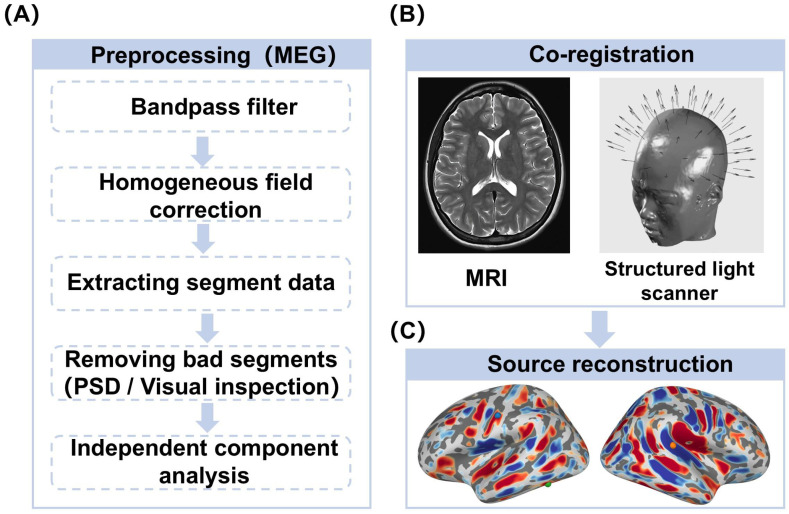
Data preprocessing and source localization. (**A**) Preprocessing. The OPM-MEG data were filtered, bad track and segment removed, and subjected to ICA. (**B**) Co-registration. The position and orientation of the arrows represented the position and orientation of the sensor relative to the participant’s MRI. (**C**) Source reconstruction. Based on registration, established algorithms were combined to localize the physiological sources of pattern similarity changes.

For OPM-MEG data, trials in which the participant made incorrect key presses were first removed to ensure the accuracy of the behavioral data. Subsequently, 1–30 Hz bandpass filter was applied (Figure 5A), and uniform field correction was applied to eliminate environmental interference. Abnormal channels were detected through power spectral density analysis and removed after manual confirmation. During the trial segmentation phase, data segments from 100 ms pre-stimulus to 1000 ms post-stimulus were extracted to form the epochs required for analysis. To ensure data quality, epochs containing obvious artifacts were again identified and removed through power spectral analysis and visual inspection. Finally, independent component analysis (ICA) was used to separate and remove residual physiological and technical artifacts, and the data was downsampled to 128 Hz.

The EEG data preprocessing process remained largely consistent with that of MEG, with appropriate adjustments to address electrophysiological signal characteristics. In addition to the same trials selection and bandpass filtering, a whole-brain average reference approach was used to enhance signal-to-noise ratio. To effectively address eye movement artifacts, “FP1” and “FP2” were labeled as EOG channels, and ICA was used to specifically remove electrooculographic and myoelectric interference. Visual inspection was also performed after epochs segmentation, with an additional baseline correction step (using the 100 ms pre-stimulus as the baseline). Finally, the data was further cleaned using ICA decomposition and downsampled to 128 Hz.

### 3.5. Co-Registration

T1-weighted structural MRI images of the participants were acquired using a Siemens 3-T scanner (Siemens Medical Solutions, Erlangen, Germany) for subsequent registration (scan parameters: TR, 2200 ms; TE, 3.37 ms; TI, 1050 ms; FA = 7°; FOV = 256 × 256 mm; voxel size = 1.0 mm × 1.0 mm × 1.0 mm). First, Freesurfer (version 7.4.1, Athinoula A. Martinos Center for Biomedical Imaging, Charlestown, MA, USA; available online: https://surfer.nmr.mgh.harvard.edu/ (accessed on 23 June 2026)) was used to reconstruct the MRI data, generating a scalp surface model containing 50,000 vertices. The participant’s head was then scanned in three dimensions using a structured light scanner (Occipital Inc., San Francisco, CA, USA). This scanned point cloud was then segmented using region growing, dividing the scanned point cloud into the face and helmet. Next, the 3D rigid helmet model and helmet point cloud were coarsely and finely registered using the random sampling consistency initial registration algorithm and the iterative closest point (ICP) algorithm, respectively. The nose tip was located based on the participant’s scan results and MRI images, and the nose region was extracted. The ICP algorithm was also used to align the face scan point cloud with the MRI image. Since the position and orientation of the sensors relative to the helmet were known during the rigid helmet design, the position and orientation of the sensors relative to the participant’s MRI were determined after these two registrations [26]. On this basis, we performed source localization on the OPM-MEG and EEG data. First, we aligned the three-dimensional head information obtained by the structured light scanner with the MRI data to obtain the position and orientation information of the sensors relative to the MRI structural image for each participant during the experiment (Figure 5B). Then, we used the head model and sensor position to calculate the forward solution using MNE-Python (version 1.6, open-source software; available online: https://mne.tools/ (accessed on 23 June 2026)), and further estimated the anatomical source and source activation time series through dynamic statistical parameteric mapping (Figure 5C).

### 3.6. Statistical Tests

To compare the time windows in which TRF-RSA and Tra-RSA pattern similarity showed significant differences, we performed the Wilcoxon rank-sum test for pattern similarity across all stimuli under TRF-RSA and Tra-RSA, with a significance level of 0.05. Furthermore, we performed the Wilcoxon rank-sum tests for pattern similarity within the six categories. Separate tests were then performed to examine differences between animate and inanimate sounds, with a significance level of 0.05. All these tests were corrected for multiple comparisons using the false discovery rate (FDR), and significant results and corresponding *p*-values were reported. In this study, the MNE-Python toolbox was utilized to map the sensor-space TRF weight matrices to the source space. First, based on the aforementioned co-registration results, we computed the individual forward Boundary Element Model (BEM). We then applied the dynamic statistical parametric mapping (dSPM) inverse algorithm to project each participant’s sensor-level TRF weight matrix onto their individual cortical source space. Subsequently, a spatial morphing procedure was used to project the TRF estimates obtained for each participant onto a common standard brain template. Next, a group-level t-test was performed on all vertices across the whole brain at each time lag, and FDR correction was applied for multiple comparisons. The significance thresholds were set at 0.05, 0.01, and 0.001, respectively, to precisely identify the significant feature-encoding brain regions involved in semantic processing. Finally, the spatiotemporal dynamic characteristics of the activated cortical regions were visualized using the MNE-Python visualization tools.

## 4. Results

### 4.1. Dynamic Changes in Similarity of Neural Response Patterns

**Figure 6:**
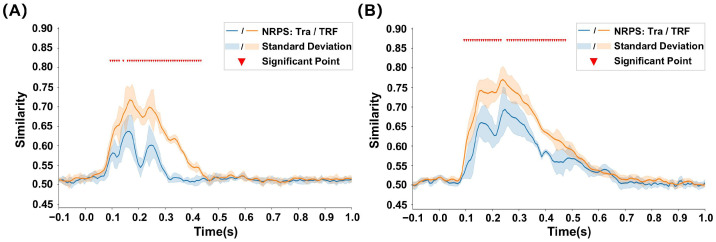
Time course of pattern similarity. The curve represented the mean similarity value at each time point, reflecting the temporal change in pattern similarity. Light shading indicated standard deviation, and red inverted triangles marked moments when TRF-RSA was significantly higher than Tra-RSA. (**A**) MEG-NRPS time series. The two conditions showed significant differences between 90 and 435 ms post-stimulus (Wilcoxon rank-sum test, *p* = 0.05). (**B**) EEG-NRPS time series. The two conditions showed significant differences between 95 and 475 ms post-stimulus (Wilcoxon rank-sum test, *p* = 0.05).

To investigate the fine-grained dynamics of NRPS during speech processing, we performed TRF-RSA and compared its performance directly with Tra-RSA. Crucially, both OPM-MEG and EEG modalities exhibited consistent temporal patterns, demonstrating the robustness of the findings across different recording techniques. Specifically, MEG results showed that NRPS remained relatively stable, maintaining a level of approximately 0.52 during the pre-stimulus period up to 70 ms post-stimulus (Figure 6A). Notably, following this baseline period, the NRPS exhibited a sharp increase, reaching 0.61 at 90 ms, at which point TRF-RSA was significantly higher than Tra-RSA (0.57). In terms of peak dynamics, the two measures manifested their primary peaks at 167 ms (TRF-RSA: 0.72) and 160 ms (Tra-RSA: 0.64), respectively. Subsequently, after a transient decline to 0.67 (TRF-RSA, 215 ms) and 0.55 (Tra-RSA, 207 ms), a prominent secondary wave emerged. This secondary response peaked at 240 ms (TRF-RSA: 0.70) and 248 ms (Tra-RSA: 0.60), marking a distinct biphasic pattern before both signals gradually decayed. Statistical analysis revealed that the pattern similarity yielded by TRF-RSA was significantly higher than that of Tra-RSA across a sustained time window from 90 to 435 ms post-stimulus. Beyond this window, no statistical differences between the two methods were further detected (TRF-RSA: 0.53; Tra-RSA: 0.51), with both NRPS tracks eventually converging back to the initial baseline of approximately 0.52.

In parallel, the EEG recordings exhibited a temporal trajectory consistent with MEG (Figure 6B). Specifically, the NRPS remained stable at approximately 0.51 from the pre-stimulus period up to 78 ms post-stimulus. Mirroring the MEG dynamics, a sharp upward trend emerged immediately thereafter. By 94 ms, the TRF-RSA similarity value reached 0.57, significantly exceeding that of Tra-RSA (0.53). Both peaked at 151 ms and 159 ms, respectively, with the TRF-RSA similarity at 0.74 and the Tra-RSA similarity at 0.66. Thereafter, both curves gradually declined, with the TRF-RSA dropping to approximately 0.73 and maintaining this level until 210 ms, while the Tra-RSA dropped to 0.63 at 207 ms. A subsequent phase of reactivation peaked at 240 ms (TRF-RSA: 0.77) and 248 ms (Tra-RSA: 0.69). Afterwards, significance testing showed that TRF-RSA yielded significantly higher pattern similarity than Tra-RSA across a wide, sustained post-stimulus window from 94 to 475 ms. By 475 ms, no further statistical difference was detected between the two methods (TRF-RSA: 0.60; Tra-RSA: 0.56), and both traces ultimately returned to the baseline level of 0.51.

### 4.2. Similarity of Neural Response Patterns Within Categories

**Figure 7:**
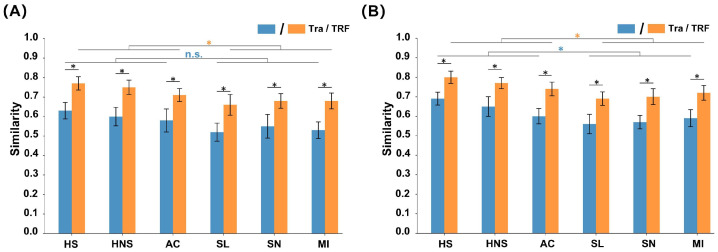
Within-category pattern similarity across different stimuli. (**A**) MEG within-category pattern similarity. Blue and orange bars represented the average pattern similarity between Tra-RSA and TRF-RSA across the 6 stimulus categories (*: *p* < 0.05, n.s.: not significant). (**B**) EEG within-category pattern similarity. Blue and orange bars represented the average pattern similarity between Tra-RSA and TRF-RSA across the 6 stimulus categories (*: *p* < 0.05).

To evaluate category-specific neural representations, we calculated the within-category pattern similarity across the six stimulus categories utilizing both Tra-RSA and TRF-RSA frameworks. In the MEG modality, Tra-RSA yielded the following NRPS values: HS: 0.63, HNS: 0.60, AC: 0.58, SL: 0.52, SN: 0.55, and MI: 0.53. Notably, the corresponding TRF-RSA measurements revealed a marked and consistent increase across all similarity metrics, reaching 0.77, 0.75, 0.71, 0.66, 0.68, and 0.68, respectively. Subsequent within-category statistical evaluations confirmed that TRF-RSA significantly enhanced pattern similarity compared to Tra-RSA across all 6 categories (FDR-corrected *p*-values: 0.042, 0.039, 0.040, 0.035, 0.031, and 0.036). A congruent pattern was manifested within the EEG modality (Figure 7B). The NRPS values obtained via Tra-RSA stood at HS: 0.69, HNS: 0.65, AC: 0.60, SL: 0.56, SN: 0.57, and MI: 0.59, whereas the TRF-RSA approach consistently elevated these metrics to 0.80, 0.77, 0.74, 0.69, 0.70, and 0.72, respectively. Parallel statistical analyses further substantiated this enhancement, demonstrating that TRF-RSA achieved significantly enhanced pattern similarity compared to Tra-RSA across all six categories (FDR-corrected *p*-values: 0.034, 0.038, 0.036, 0.033, 0.041, and 0.034).

Furthermore, we conducted a comparative analysis of NRPS between animate and inanimate sounds, aiming to evaluate the performance of the two frameworks in distinguishing the neural representation patterns associated with these broad categories. In the MEG modality (Figure 7A), the results showed that no significant NRPS difference between the two categories under Tra-RSA (*p* = 0.095), whereas TRF-RSA successfully detected enhanced pattern similarity for animate sounds relative to inanimate sounds (*p* = 0.037). Similarly, in the EEG modality (Figure 7B), while both frameworks revealed that animate sounds elicited significantly higher NRPS than inanimate sounds, TRF-RSA substantially enhanced the statistical robustness, strengthening the significance level from 0.041 (Tra-RSA) to 0.024 (TRF-RSA). Therefore, by isolating stimulus-locked neural traces and filtering out noise, TRF-RSA enhances both within-category similarity and between-category separability.

### 4.3. Source Localization of Neural Response Pattern Similarity

**Figure 8:**
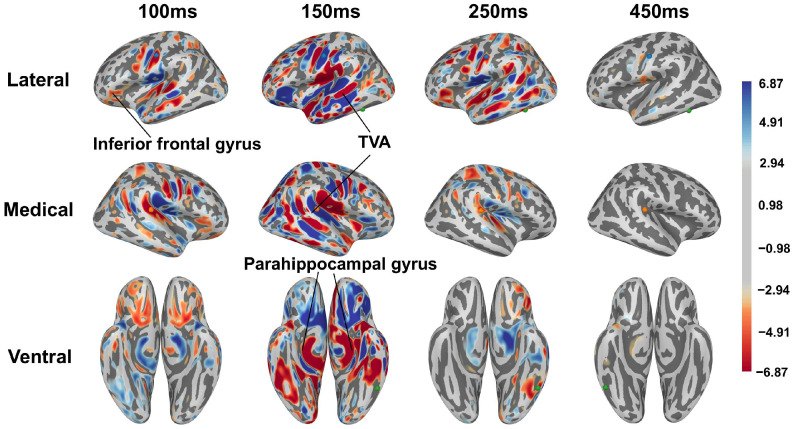
MEG source localization of pattern similarity. The results showed that HG, IFG, TVA, PG, OFG, etc. were significantly activated during sound processing.

To delineate the spatiotemporal evolution of speech encoding, we investigated the anatomical origins of pattern similarity through MEG and EEG source localization analyses. MEG results revealed significant activation of the inferior frontal gyrus (IFG), Heschl’s gyrus (HG), orbito-frontal gyrus (OFG), and parahippocampal gyrus (PG) 100 ms post-stimulus (Figure 8). As speech processing progressed to 150 ms, a prominent expansion in both cortical recruitment and signal intensity was observed. Significant activation persisted within the core network of the IFG, HG, OFG, and PG, exhibiting substantially greater spatial extent compared to 100 ms. This early activation in the primary auditory cortex and frontal regions is consistent with known neural mechanisms underlying the initial extraction of acoustic features [27]. Notably, the cortical engagement concurrently extended into high-level language and semantic networks, including the temporal voice area (TVA), inferior temporal gyrus (ITG), and the Insula. This widespread cortical orchestration at 150 ms aligns with the transition from low-level sensory representations to the integration of complex acoustic features [27,28]. At 250 ms, activation decreased in both intensity and range in all brain regions, with the PG remaining somewhat activated. No significant activation was observed at 450 ms.

**Figure 9:**
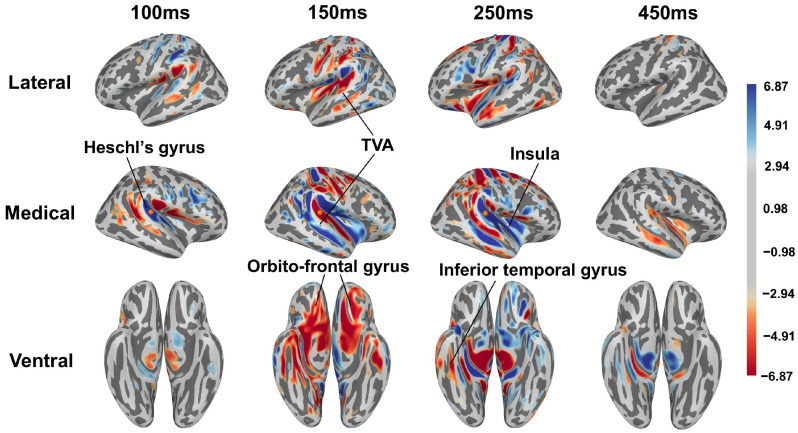
Source localization of EEG pattern similarity. The source localization results have high spatiotemporal consistency with MEG.

EEG source localization showed similar spatiotemporal distribution characteristics to MEG (Figure 9). Initial activation was observed in HG at 100 ms post-stimulus. At 150 ms, robust cortical activation was identified in the TVA, OFG, IFG, Insula, and ITG. Significant activation at 250 ms was observed in the right TVA, Insula, PG, and ITG. Except for the TVA, activation intensity and range in the remaining regions decreased compared to 150 ms. By 450 ms, significant activation was observed in the right Insula, middle temporal gyrus (MTG), and PG.

## 5. Discussion

We obtained OPM-MEG and EEG recordings during a speech discrimination task to characterize dynamic NRPS. By innovatively integrating time-lagged regression with multivariate pattern analysis, we discovered that TRF-RSA provides enhanced sensitivity for identifying neuronal representations. Furthermore, we found that TRF-RSA significantly enhanced within-category pattern similarity across the 6 types of sound stimuli, with particularly strong similarity observed for animate sounds. Source localization revealed that speech processing and discrimination involved a distributed neural network system that included not only classical language areas but also limbic brain areas.

### 5.1. Early Pattern Similarity Changes in Speech Encoding

Numerous studies in auditory cognitive neuroscience have confirmed that the human auditory cortex tracks the speech envelope through neural oscillatory synchronization [6,7]. This acoustic-neural tracking serves as a core, foundational neural mechanism for perceiving continuous speech streams [18,29]. This study used multimodal neuroimaging technologies, combined with time-lagged regression and RSA, to examine the dynamic time course of NRPS during speech encoding. The results showed that TRF-RSA was more sensitive than Tra-RSA, showing significantly higher pattern similarity across multiple time windows. The high consistency observed between OPM-MEG (90–435 ms post-stimulus) and EEG (95–475 ms post-stimulus) strongly suggestd that TRF-RSA effectively improved the resolution of neural representations. Pattern similarity began to rise sharply around 70 ms and exhibited significant divergence by approximately 90 ms. These results were consistent with the findings of Murray et al., whose auditory evoked potential studies had shown early processing of complex sound categories beginning at 70 ms post-stimulus [27]. Further supporting evidence demonstrated that auditory information could be analyzed and integrated into consciously perceivable representations within 80–120 ms post-stimulus [30]. These findings suggested that neural pattern similarity influenced early speech encoding stages. Numerous studies suggest that during the early stages of sound processing (e.g., 90–160 ms in this study), the brain establishes representational synchronization between neuronal populations, leading to increased pattern similarity. This is consistent with the results of Correa et al. [31] that activity synchronization exists during the early stages of perception. This early enhancement in pattern similarity essentially reflects the representational synchronization among neural populations. The ability to precisely capture and quantify this synchronization mechanism is of significant importance. Current neuroscientific research actively explores the use of external rhythmic stimulation (such as acoustic driving) to shape the temporal structure of brain activity, serving as a promising neuromodulation tool for improving cognitive functions like memory or sleep [32,33]. Source localization results during this period showed activation primarily in the HG, IFG, and OFG regions. This finding is aligned with the early processing network proposed by Remedios et al., suggesting that neural activity recorded during this timeframe originates primarily from auditory areas of the temporal lobe and the frontal cortex [34].

By integrating stimulus features with temporal dependencies, TRF-RSA effectively isolates and extracts stimulus-evoked neural signals, thereby enabling fluctuations in pattern similarity to be more precisely attributed to underlying sensory processing. This is crucial in everyday environments because speech is often embedded in complex acoustic backgrounds, requiring listeners to actively separate speech from background sounds for selective attention and processing [35]. Consequently, TRF-RSA expands the statistical distance and separability between distinct categorical configurations within the representational space. These methodological advantages hold implications for future research aiming to analyze the dynamic encoding differences or synchronicity of various stimulus features.

### 5.2. Differences in NRPS Between Vocalizations of Animate and Non-Animate

TRF-RSA acts as a “noise filter” by elevating the computation of similarity from the neural signal space to the TRF weight space. Since weights are derived via time-lagged regression between neural responses and stimulus features, TRF-RSA extracts stimulus-locked neural activity, thereby effectively filtering out task-irrelevant noise. Consequently, TRF-RSA upgrades the analytical perspective from a static comparison of raw signal intensities to a dynamic comparison of the encoding process. We found that pattern similarity remained high for 150–250 ms post-stimulus, and M/EEG source localization consistently showed significant activation of the TVA region within this timeframe. This region, located along the bilateral middle/anterior superior temporal sulcus and superior temporal gyrus (STS/STG), is in close proximity to core auditory areas (approximately two to three synapses) and contains a large number of sound-sensitive cells [36]. The TVA has been reported to respond more strongly to sounds of the same species than to other sound categories [37]. Numerous studies indicate that activation of the TVA 100–250 ms after a sound stimulus reflects rapid discrimination of the input stimulus [28,38]. This phenomenon is also supported by many MEG studies: through systematic analysis of the participants’ response characteristics to different sound stimuli, it was found that the brain activity recorded by the temporal lobe electrodes began to show obvious stimulus specificity during 120–150 ms, and reached the activation peak during 200–250 ms [39,40].

Our results demonstrate that, in the MEG modality, there are significant differences in the representational similarity between animate and inanimate sounds detected by TRF-RSA compared to Tra-RSA. Furthermore, the findings show that TRF-RSA significantly enhanced pattern similarity across all six distinct stimulus categories under both MEG and EEG. This supports the ability of TRF-RSA to enhance sensitivity to neural representations across diverse acquisition modalities and stimulus types. For humans and many other species, the most critical category of sounds is species-specific vocalizations. These findings initially support the idea that species-specific vocalizations serve as highly effective stimuli, evoking stronger neural responses compared to less relevant sounds [41,42], resulting in more consistent representational patterns [43,44]. Cross-species studies consistently show that organisms exhibit significant neural response preferences toward conspecific or animate vocalizations, which has been observed in several primate species [45,46]. At the same time, the loss of this sound preference is likely an important characteristic of specific neurodevelopmental disorders or acquired brain injury. For instance, in patients with Phonagnosia, the ability to recognize speech content (linguistic information) is preserved, yet the recognition of the speaker’s identity (paralinguistic information) is impaired. This suggests functional specificity in the underlying neural basis of voice processing [47]. Crucially, research on Autism Spectrum Disorder (ASD) has demonstrated that the STS region in patients often fails to exhibit the characteristic enhanced activation to human voices observed in neurotypical individuals. This implies that voices may be non-specifically encoded in their neural representations [48]. The TRF-RSA holds significant potential in the early diagnosis of social communication deficits. By quantifying whether an individual demonstrates a typical animate sound preference, we can investigate early neural coding deficits in the social information processing pathway of children with ASD or patients with Phonagnosia. Importantly, compared to traditional assessments relying solely on behavioral outcomes, this neuro-encoding-based metric offers the ability to precisely localize the deficit: whether it originates in the early sensory encoding stage or the later stage of high-level cognitive integration. This provides possible supporting evidence for the early diagnosis and intervention of social communication impairments.

### 5.3. Distributed Neural Network System Activation

Both MEG and EEG source localization results reveal activation in multiple brain regions during speech encoding. The STS/STG, as core components of the TVA, are crucial hubs for speech encoding [49]. Activation in the TVA began as early as 150 ms post-stimulus, coinciding with the critical time window for speech encoding [50]. More broadly, functional neuroanatomical evidence further suggests that the HG, MTG, ITG, OFG, and TVA in this study constitute a core network for speech processing [51,52]. Notably, we leveraged the high sensitivity and spatiotemporal resolution of TRF-RSA. This approach not only confirmed the involvement of the classic speech processing system but also reveal activation in regions such as the PG and Insula. Clinical studies demonstrate that the PG is located in the medial temporal lobe, which maintains close functional connectivity with the auditory cortex, and participates in memory encoding through hippocampal circuits [53,54]. Growing evidence suggests that hearing impairment is a factor for cognitive decline and dementia. In this association, the PG plays a critical role as the key functional interface connecting central auditory processing with higher-level cognitive functions. Recent studies have shown that PG activation is not only related to the hearing threshold but also mediates the relationship between central hearing and memory function. Furthermore, its connections within the limbic system are crucial in the genesis of tinnitus and the modulation of emotional stress [55]. In the context of an aging population, TRF-RSA is poised to track the neural representational changes in patients with hearing loss, specifically by monitoring for abnormal activity patterns in critical nodes like the PG. This could help in the early identification of individuals at high risk of Alzheimer’s disease or other dementias before behavioral cognitive impairments become apparent. Although the Insula has not been included in traditional speech network models and its underlying neural basis is poorly understood, our results are consistent with previous human imaging and lesion studies, suggesting that the Insula is involved in complex speech processing [34]. Traditional multivariate decoding approaches frequently overlook the weak but sustained stimulus-driven activity within these limbic and deep structures. Crucially, TRF-RSA incorporated these regions, typically associated with memory and emotion, into the neural representation of speech encoding. TRF-RSA improves the sensitivity of activation pattern detection by effectively filtering out noise and extracting stimulus-driven patterns. Therefore, this method provides a new avenue for future research aimed at revealing changes in representational similarity within the limbic system and deep brain structures.

### 5.4. The Potential for Expansion and Application of Multivariate Decoding Techniques

Speech comprehension is a highly complex cognitive function that requires the brain to process continuous acoustic signals rapidly and online to precisely extract structure and meaning. Although the spatial patterns of neuronal populations reflect considerable detail regarding speech perception, these representational details are often difficult to precisely discern due to the complexity of encoding and variations in the stimulus environment and physiological state. Furthermore, the optimal way to encode stimulus information may also change over time [56]. The TRF-RSA overcomes the limitations of traditional methods relying on discrete, static stimuli by establishing a time-lagged mapping between continuous natural stimuli and neural responses, expanding the scope of multivariate decoding techniques.

Firstly, this flexibility allows it to be applied to the assessment of specific clinical populations. Many neurodevelopmental disorders (e.g., dyslexia and developmental language disorder) are linked to deficits in the brain’s temporal tracking of the speech envelope [57]. TRF-RSA can utilize natural stimuli (such as cartoons and stories) to assess the accuracy of this temporal encoding without requiring complex cooperation from the subjects. This is particularly beneficial for investigating populations challenging to assess with traditional experimental paradigms, such as infants and children with attention deficits or elderly individuals with cognitive impairments [58,59]. Furthermore, TRF-RSA can furnish objective neural metrics for auditory prostheses. Current clinical audiology diagnostics (such as pure-tone audiometry) often fail to fully reflect a patient’s communication difficulties in real-world noisy environments (the cocktail party effect). Pattern similarity may serve as an objective neural surrogate measure for speech intelligibility. This opens possibilities for future smart hearing aids and cochlear implants [60]. For example, although they can compensate for the typical decreased audibility by amplifying the signal, it has been demonstrated that this compensation cannot fully restore speech understanding [61]. By real-time decoding of the brain’s neural tracking quality of target speech, noise reduction algorithms or gain parameters can be automatically adjusted, enabling adaptation to complex acoustic environments. Finally, TRF-RSA possesses high extensibility because it can extract complex neural responses to numerous stimulus features from neural activity elicited by continuous, natural stimulation (applicable to mappings across various stimulus types, features, and modalities). Beyond diagnosis and early identification, it will also facilitate the objective evaluation of neuroplasticity changes induced by interventions (such as auditory training or cochlear implantation). By capturing these complex, dynamic neural representations under natural conditions, TRF-RSA provides a new reference for realizing precise individualized auditory rehabilitation and personalized clinical application.

## 6. Limitations and Prospects

The experimental OPM-MEG and EEG data were collected separately. Although this independent recording method is relatively simple and can be optimized for the characteristics of different technologies, asynchronous recording may lead to differences in conditions such as task state, environment, and participant physiological state between the different modalities. Given the current development trend of multimodal synchronous acquisition technology, future research will gradually adopt synchronous recording schemes. Concurrently, further enriching sample diversity in terms of ethnicity, age, cultural background, and potential genetic variations represents an important direction for future research. This expansion will facilitate the cross-validation of the generalizability and robustness of our findings across a broader population. On the other hand, the complex spatiotemporal patterns of brain activity increase the demand for computational resources to decode speech using RSA. Therefore, in the future, we will not only adopt more effective dimensionality reduction strategies and pattern recognition methods, but also focus on developing efficient parallel computing architectures to optimize the computational process and further improve research efficiency. This is essential for translating TRF-RSA into a fast, integrated, portable clinical assessment tool that can be deployed widely.

## 7. Conclusions

This study modeled the response characteristics of the human brain during speech perception by integrating both stimulus features and neural signals. By combining time-lagged regression with multivariate analysis, we established TRF-RSA as a more discriminative and sensitive representation analysis tool. The results demonstrate that TRF-RSA overcomes the limitations of traditional RSA, effectively capturing stimulus-driven neural activity and significantly enhancing pattern sensitivity during speech encoding. Furthermore, the study not only amplified within-category pattern similarity for speech stimuli but revealed stronger similarities between biological sounds, suggesting a preference for these semantic categories. At the network level, source localization confirmed the involvement of the classical speech network while also revealing extended activation in limbic and deep brain regions, pointing toward a more distributed neural architecture for speech perception. Furthermore, our analysis revealed activation in limbic and deep brain structures. By modeling the mapping between stimulus features and neural responses, TRF-RSA dynamically captures the spatiotemporal patterns of stimulus-evoked brain activity, thereby improving the sensitivity of characterizing neural representations during speech encoding. These findings suggest that this framework serves as a powerful analytical tool that not only elucidates the spatiotemporal dynamics of speech processing, but also offers a more nuanced perspective on the distributed neural mechanisms underlying language comprehension.

## Data Availability

The data and code generated during the current study are available from the corresponding author on reasonable request.
